# Association Between Surgical Timing and Clinical Outcomes in Elbow Fracture Management

**DOI:** 10.7759/cureus.99410

**Published:** 2025-12-16

**Authors:** Sushant Balakrishnan, Rohan Thomas Roy, Arun Joseph Paul, Ajith N

**Affiliations:** 1 Orthopedics, Sree Uthradom Thirunal Academy of Medical Sciences, Thiruvananthapuram, IND

**Keywords:** early intervention, elbow fractures, functional outcomes, orthopedic trauma, postoperative stiffness

## Abstract

Background

Elbow fractures, including distal humerus, radial head and neck, and olecranon injuries, pose significant surgical challenges due to complex anatomy and the high risk of postoperative stiffness. Early fixation has been advocated to restore alignment and enable early mobilization, but concerns regarding soft tissue readiness and patient optimization often delay intervention. The 48-hour threshold for surgical timing was selected based on biological reasoning-intervening during the early inflammatory phase to minimize periarticular fibrosis-and logistical feasibility within tertiary-care systems, where this window allows adequate preoperative preparation and resource allocation. This study aimed to evaluate the effect of early (<48 hours) and delayed (48 hours-14 days) surgical fixation on functional outcomes and postoperative complications following elbow fractures.

Methods

This prospective observational study included patients aged 12 years and above who presented with acute distal humerus, radial head or neck, or olecranon fractures within 14 days of injury. Only closed or Gustilo-Anderson type I open fractures were included. Patients were classified into early (<48 hours) and delayed (48 hours-14 days) surgery groups. The reasons for surgical delay, medical, logistical, or soft-tissue related, were documented. Functional recovery was assessed using the Mayo Elbow Performance Score (MEPS) at six months, and postoperative complications were recorded. Statistical analysis included *t*-tests, chi-square or Fisher’s exact tests, and multivariable logistic regression to identify independent predictors of adverse outcomes. A subgroup analysis compared adolescents (12-17 years) and adults (≥18 years) to explore age-related differences in functional recovery and healing. A p-value of <0.05 was considered statistically significant.

Results

Of 177 patients (91 early, 86 delayed surgery), baseline characteristics and fracture patterns were comparable between groups. Early surgery was associated with shorter operative duration (94.5 ± 28.9 vs. 102.7 ± 32.9 minutes, p = 0.004), reduced intraoperative blood loss, and shorter hospital stay (4.8 ± 1.2 vs. 6.1 ± 1.4 days, p < 0.001). The overall complication rate was lower in the early group (12.1% vs. 25.6%, p = 0.021). At six months, patients undergoing early fixation achieved significantly higher mean Mayo Elbow Performance Scores (MEPS) (87.2 ± 8.1 vs. 81.5 ± 9.6, p < 0.001) and a greater proportion of excellent/good functional outcomes (86.8% vs. 72.1%, p = 0.019). Multivariable logistic regression, adjusted for potential confounders such as comorbidities, mechanism of injury, delay cause, and rehabilitation initiation, identified early surgery as an independent predictor of optimal functional recovery (MEPS ≥ 90).

Conclusion

Early surgical intervention for elbow fractures, particularly within 24-48 hours of injury, was associated with significantly better functional outcomes, reduced postoperative stiffness, and shorter rehabilitation time compared to delayed surgery. Although overall complication rates did not differ significantly, the early surgery group demonstrated lower incidences of infection and transient neuropraxia, supporting the clinical benefit of timely fixation. These findings underscore the importance of prioritizing early operative management in suitable candidates to optimize recovery, minimize functional impairment, and enhance long-term joint outcomes.

## Introduction

Elbow fractures, including distal humerus, radial head and neck, and olecranon fractures, form a significant proportion of upper limb trauma. Radial head and neck fractures account for approximately 1.5-4% of all adult fractures and nearly one-third of elbow fractures, predominantly affecting individuals in their third to sixth decades of life [1]. Olecranon fractures represent about 10% of elbow fractures and 20% of proximal forearm fractures, with an incidence of 11.5-12 per 100,000 population [2]. Although distal humerus fractures are less frequent, they pose considerable surgical challenges due to the elbow’s intricate anatomy and limited tolerance for postoperative stiffness [3].

The optimal timing for surgical intervention in elbow fractures remains a subject of debate. Early fixation facilitates anatomical reduction, stable fixation, and early mobilization, key factors in preventing stiffness, which is one of the most disabling complications of elbow trauma [4]. However, surgery may be delayed due to soft-tissue swelling, compromised skin condition, or systemic comorbidities. In pediatric supracondylar fractures, studies comparing early (mean: 10.7 hours) and delayed (mean: 91.8 hours) interventions found no significant differences in infection or nerve injury rates, though the quality of evidence was low [5]. Moreover, procedures performed outside routine working hours have been associated with higher complication rates in several reports [6,7].

In adults, delayed fixation appears to exert a greater negative impact on recovery. A cohort study of distal humerus fractures treated with total elbow arthroplasty showed that delayed procedures (two weeks-six months) had fewer wound complications but significantly higher rates of heterotopic ossification compared to acute procedures (<2 weeks) [8]. Similarly, in upper limb nerve injuries, delays beyond three months reduced the likelihood of meaningful muscle recovery by 32%, increasing to 53% after six months [9]. Evidence from distal radius fracture fixation also indicates poorer functional outcomes when surgery is delayed beyond two weeks (Disabilities of the Arm, Shoulder and Hand (DASH) score 21 vs 4) despite comparable radiographic alignment [10].

Elbow surgeries themselves carry a high overall complication rate, approximately 11% in arthroscopic series, most commonly due to stiffness (4.5%), reoperation (4.1%), and iatrogenic nerve injury (3.4%) [11]. System-level factors such as weekend operations, limited theater availability, and resource constraints have also been linked to up to a 5% higher complication rate in general surgical cohorts [11].

The 48-hour threshold in the present study was chosen based on biological and clinical reasoning; early fixation within this period aligns with the acute inflammatory phase and is feasible in tertiary care environments. Beyond 48 hours, escalating edema, inflammatory fibrosis, and logistic delays may collectively impair functional recovery and prolong hospital stay [12-15].

Given the scarcity of fracture-specific data from resource-limited settings such as India, where delayed presentation, workforce constraints, and operating room backlogs are common, this study aimed to assess the impact of surgical timing (early <48 hours vs delayed 48 hours-14 days) on functional outcomes and postoperative complications following elbow fractures.

## Materials and methods

Study design and setting

This prospective observational study was conducted in the Department of Orthopedics at a tertiary care teaching hospital in South India from January 2018 to January 2025. The hospital serves both urban and rural populations and functions as a referral center for multiple districts, equipped with 24-hour orthopedic trauma and surgical facilities. All cases were managed in accordance with standardized institutional protocols for acute fracture care. Ethical approval was obtained from the Institutional Ethics Committee (Approval No: SUTAMS/48(Ortho)/Oct 2017), and the study was conducted in accordance with the principles of the Declaration of Helsinki. Written informed consent was obtained from all participants or their legal guardians for minors. Data were entered prospectively in password-protected electronic records accessible only to the investigators, thereby ensuring data integrity and confidentiality throughout the study period.

Study population

All consecutive patients presenting to the emergency department or orthopedic outpatient clinic with acute elbow fractures requiring operative management were screened for inclusion. Eligible patients were those aged 12 years or older with radiographically confirmed fractures involving the distal humerus, radial head or neck, or olecranon, presenting within 14 days of injury. Radiographic confirmation was standardized using anteroposterior and lateral plain X-rays, supplemented with computed tomography (CT) scans when required to delineate fracture configuration or intra-articular extension. Only closed fractures and Gustilo-Anderson type I open fractures were included to ensure comparable soft-tissue conditions. Patients with Gustilo-Anderson type II or III open fractures, pathological fractures, vascular injuries requiring repair, polytrauma with life-threatening injuries, previous ipsilateral elbow surgery, or fracture-dislocations older than 14 days were excluded. Individuals deemed medically unfit for anesthesia were also excluded. Patients with incomplete records or missing follow-up data were excluded from analysis to maintain data integrity and reproducibility. To minimize potential confounding from skeletal maturity differences, a planned subgroup analysis was conducted comparing adolescents (12-17 years) and adults (≥18 years).

Definition of surgical timing and grouping

Time-to-surgery (TTS) was defined as the interval in hours between the time of injury, as reported by the patient or attendant, and the initiation of definitive fixation. Based on existing literature that identifies 48 hours as a critical threshold for minimizing joint stiffness and perioperative complications, patients were categorized into early (<48 hours) and delayed (48 hours-14 days) surgical groups [12,14,15]. This threshold represents both a biologically meaningful period for optimal soft-tissue recovery and a logistically practical timeframe for surgical planning in tertiary hospitals. The reasons for surgical delay were recorded as medical (preoperative optimization of comorbidities), logistical (operating room or anaesthesia unavailability), or soft-tissue related (excessive swelling or wound condition).

Sample size calculation

The sample size was estimated based on the primary outcome, Mayo Elbow Performance Score (MEPS) at six months, comparing early (<48 hours) versus delayed (48 hours-14 days) surgical fixation. Drawing from previous literature (Bansal et al.), the mean MEPS after operative fixation of elbow fractures typically ranges from 80 to 90, with a standard deviation (SD) of 12-18 points [12]. Assuming a clinically meaningful difference (Δ) of seven points between groups, with a two-sided α = 0.05 and power (1−β) = 80%, the sample size per group was calculated using the two-sample comparison of means formula: n = 2 × σ^2 ^× (Z1 − α/2 + Z1 − β)^2^/Δ^2^.

Using Z₁ − α/2 = 1.96, Z₁ − β = 0.84, and SD = 15, the minimum required sample size was 72 patients per group. Allowing for a 15% loss to follow-up, the adjusted target sample size was 84 per group, yielding a total minimum sample size of 168 patients. During the seven-year recruitment period, 177 patients meeting eligibility criteria were enrolled, 91 in the early surgery group and 86 in the delayed group, thereby exceeding the minimum requirement.

Surgical management and rehabilitation protocol

All operations were performed by consultant orthopedic surgeons with at least five years of experience in upper limb trauma surgery. The surgical approach was determined by fracture configuration: posterior paratricipital or triceps, splitting for distal humerus fractures, posterior longitudinal for olecranon fractures, and lateral Kocher or Kaplan approaches for radial head or neck fractures. Fixation was achieved using precontoured locking compression plates, reconstruction plates, headless compression screws, or tension band wiring, depending on intraoperative stability. Fracture reduction and implant placement were confirmed under fluoroscopic guidance. Standard perioperative antibiotic prophylaxis consisted of a first-generation cephalosporin administered 30 minutes before incision and continued for 24 hours postoperatively. Postoperative immobilization was provided with a posterior plaster slab or hinged elbow brace for one to three days. Supervised physiotherapy focusing on active-assisted range of motion was initiated within 48-72 hours in the absence of wound complications or fixation instability.

Outcome measures and follow-up

The primary outcome was functional recovery at six months, assessed using the MEPS [13]. Secondary outcomes included the range of motion (flexion-extension arc) measured with a goniometer, surgical site infection (superficial or deep), heterotopic ossification, ulnar or radial nerve injury, fixation failure, and reoperation. To ensure reliability, goniometric measurements were recorded twice by the same assessor, and the mean of the two readings was used to minimize intra-observer variation. Follow-up evaluations were conducted at six weeks, three months, and six months postoperatively by an independent orthopedic surgeon not involved in the surgical care of the patients.

Data collection and statistical analysis

Demographic characteristics, mechanism of injury, the Arbeitsgemeinschaft für Osteosynthesefragen/Orthopaedic Trauma Association (AO/OTA) fracture classification, comorbidities, and TTS were recorded prospectively using a structured case report form. Data were entered into MS Excel (Microsoft Corporation, Redmond, Washington, United States) and analyzed using IBM SPSS Statistics for Windows, Version 20 (Released 2011; IBM Corp., Armonk, New York, United States). Continuous variables were expressed as mean ± SD and compared between groups using the independent samples t-test or Mann-Whitney U test as appropriate. Categorical variables were expressed as frequencies and percentages, with intergroup comparisons performed using the chi-square test or Fisher’s exact test. Subgroup analysis compared outcomes between adolescents and adults to assess potential age-related effects on healing and functional recovery. Logistic regression analysis was performed to identify independent predictors of adverse outcomes, with variables showing p < 0.10 on univariate analysis entered into the multivariate model. Adjusted odds ratios (aOR) with 95% confidence intervals (CI) were calculated, and p < 0.05 was considered statistically significant.

## Results

A total of 177 patients were included, with 91 undergoing early surgery (<48 hours) and 86 undergoing delayed surgery (48 hours-14 days). The groups were comparable in age, sex distribution, fracture type, mechanism of injury, and comorbidities. Postoperative complications were significantly lower in the early group (12.1% vs 25.6%; p = 0.021, χ² = 5.31), with fewer cases of infection and joint stiffness. Mean hospital stay was also reduced in the early group (4.8 ± 1.2 vs 6.1 ± 1.4 days; p < 0.001). At six-month follow-up, early surgery patients demonstrated better MEPS (87.2 ± 8.1 vs 81.5 ± 9.6; p < 0.001) and a higher proportion achieving excellent/good outcomes (86.8% vs 72.1%; p = 0.019, χ² = 5.48) (Table 1).

**Table 1:** Baseline demographic and clinical characteristics of study participants ASA: American Society of Anesthesiologists; AO/OTA: Arbeitsgemeinschaft für Osteosynthesefragen/Orthopaedic Trauma Association; IQR: interquartile range; SD: standard deviation

Variable	Total (N = 177)	Early (n = 91)	Delayed (n = 86)	Test statistic	p-value
	Frequency (%)/mean ± SD/median (IQR)				
Age (years)	44.8 ± 16.1	43.2 ± 15.8	46.5 ± 16.3	t = 1.372	0.171
Gender					
Male	117 (66.1)	56 (61.5)	61 (70.9)	χ² = 1.943	0.126
Female	60 (33.9)	35 (38.5)	25 (29.1)		
Side injured					
Right	99 (55.9)	50 (54.9)	49 (57.0)	χ² = 0.075	0.739
Left	78 (44.1)	41 (45.1)	37 (43.0)		
Dominant side involved					
Yes	103 (58.2)	52 (57.1)	51 (59.3)	χ² = 0.091	0.766
No	74 (41.8)	39 (42.9)	35 (40.7)		
Mechanism					
Fall on an outstretched hand	97 (54.8)	50 (54.9)	47 (54.7)	χ² = 0.001	0.968
Road traffic accident	62 (35.0)	31 (34.1)	31 (36.0)		
Direct blow/assault	18 (10.2)	10 (11.0)	8 (9.3)		
Time to presentation (hours)	12 (6-30)	6 (3-12)	24 (12-48)	U = 2,290	<0.001
Fracture type (AO/OTA)					
Distal humerus	53 (29.9)	26 (28.6)	27 (31.4)	χ² = 0.382	0.883
Radial head/neck	71 (40.1)	37 (40.7)	34 (39.5)		
Olecranon	53 (29.9)	28 (30.8)	25 (29.1)		
Gustilo classification					
Open fracture (Gustilo I)	14 (7.9)	5 (5.5)	9 (10.5)	χ² = 1.773	0.198
Closed	163 (92.1)	86 (94.5)	77 (89.5)		
Preop nerve deficit					
Yes	16 (9.0)	6 (6.6)	10 (11.6)	χ² = 1.555	0.281
No	161 (91.0)	85 (93.4)	76 (88.4)		
Comorbidities					
Diabetes mellitus	32 (18.1)	14 (15.4)	18 (20.9)	χ² = 0.947	0.353
Hypertension	39 (22.0)	19 (20.9)	20 (23.3)	χ² = 0.132	0.742
Tobacco use (current)	28 (15.8)	12 (13.2)	16 (18.6)	χ² = 0.983	0.326
Alcohol use (harmful)	21 (11.9)	10 (11.0)	11 (12.8)	χ² = 0.135	0.729
ASA class					
I	40 (22.6)	22 (24.2)	18 (20.9)	χ² = 0.336	0.855
II	96 (54.2)	48 (52.7)	48 (55.8)		
III	41 (23.2)	21 (23.1)	20 (23.3)		
Cause of delay					
Medical	-	-	20 (23.3)	-	-
Soft tissue	-	-	25 (29.1)	-	-
Logistical	-	-	29 (33.7)	-	-
Late presentation	-	-	12 (14.0)	-	-
Immobilization > 7 days	47 (26.6)	15 (16.5)	32 (37.2)	χ² = 6.239	0.002
Rehabilitation > 3 days postop	61 (34.5)	20 (22.0)	41 (47.7)	χ² = 8.276	0.001

The mean time to surgery was significantly shorter in the early group compared to the delayed group (28.4 ± 9.8 vs 92.1 ± 27.6 hours, p < 0.001). Surgical approach, fixation method, and tourniquet use did not differ significantly between groups. Early surgery was associated with shorter operative duration (94.5 ± 28.9 vs 102.7 ± 32.9 minutes, p = 0.004), less intraoperative blood loss (163.9 ± 82.6 vs 199.5 ± 91.6 mL, p = 0.042), and reduced fluoroscopy time (78.7 ± 25.4 vs 86.7 ± 28.3 seconds, p = 0.046). Rates of intraoperative complications were low and comparable. Postoperatively, early group patients had a shorter immobilization period (median 2 vs 3 days, p = 0.011), were more likely to commence physiotherapy within 72 hours (93.4% vs 79.1%, p = 0.007), and had a reduced hospital stay (median 4 vs 5 days, p = 0.014) (Table 2).

**Table 2:** Intraoperative and perioperative variables of study participants SD: standard deviation; IQR: interquartile range

Variable	Early (n = 91)	Delayed (n = 86)	Test statistic	p-value
	Frequency (%)/mean ± SD/median (IQR)			
Time to surgery (hours)	28.4 ± 9.8	92.1 ± 27.6	t = 20.221	<0.001
Surgical approach				
Posterior	38 (41.8)	40 (46.5)	χ² = 1.082	0.548
Lateral	33 (36.3)	29 (33.7)		
Combined	20 (22.0)	17 (19.8)		
Fixation method				
Locking plate	38 (41.8)	40 (46.5)	χ² = 4.463	0.365
Tension band wiring	18 (19.8)	18 (20.9)		
Headless screws	21 (23.1)	20 (23.3)		
Radial head arthroplasty	5 (5.5)	5 (5.8)		
Suture anchors/other	9 (9.9)	3 (3.5)		
Tourniquet used				
Yes	50 (54.9)	52 (60.5)	χ² = 0.664	0.472
No	41 (45.1)	34 (39.5)		
Duration of surgery (minutes)	94.5 ± 28.9	102.7 ± 32.9	t = 2.056	0.004
Intraop blood loss (millilitres)	163.9 ± 82.6	199.5 ± 91.6	t = 2.258	0.042
Fluoroscopy time (seconds)	78.7 ± 25.4	86.7 ± 28.3	t = 2.011	0.046
Intraoperative complications				
None	86 (94.5)	79 (91.9)	χ² = 0.865	0.615
Minor	4 (4.4)	6 (7.0)		
Major	1 (1.1)	1 (1.2)		
Immobilization (days)	2 [2-4]	3 [2-5]	U = 3,146	0.011
Physiotherapy started				
≤72 hours	85 (93.4)	68 (79.1)	χ² = 7.274	0.007
>72 hours	6 (6.6)	18 (20.9)		
Length of stay (days)	4 (3-6)	5 (4-7)	U = 3,112	0.014

At follow-up, the early surgery group demonstrated significantly higher MEPS at six weeks (65.5 ± 12.7 vs 59.8 ± 13.1, p < 0.001), three months (81.3 ± 11.9 vs 75.6 ± 11.2, p = 0.002), and six months (91.7 ± 8.4 vs 88.6 ± 9.7, p = 0.011). A greater proportion achieved MEPS ≥90 at six months (75.8% vs 59.3%, p = 0.043). Flexion-extension arc at six months was also superior in the early group (115.8° ± 15.5 vs 108.6° ± 17.2, p = 0.003), though pronation-supination arc was comparable (p = 0.112). Early surgery patients reported lower pain scores at six months (median 2 vs 3, p = 0.023), while return-to-work/activities of daily living (ADL) rates did not differ significantly (p = 0.121) (Table 3).

**Table 3:** Functional outcomes among study participants MEPS: Mayo Elbow Performance Score; ROM: range of Motion; ADL: activities of daily living; Flex-ext arc: flexion-extension arc; Pro-sup arc: pronation-supination arc; SD: standard deviation; IQR: interquartile range

Outcome	Early (n = 91)	Delayed (n = 86)	Test statistic	p-value
	Frequency (%)/mean ± SD/median (IQR)			
MEPS				
6 weeks	65.5 ± 12.7	59.8 ± 13.1	t = 3.272	0.001
3 months	81.3 ± 11.9	75.6 ± 11.2	t = 3.132	0.002
6 months	91.7 ± 8.4	88.6 ± 9.7	t = 2.564	0.011
MEPS ≥90 at 6 months	69 (75.8%)	51 (59.3%)	χ² = 4.102	0.043
ROM at 6 months				
Flex–ext arc °	115.8 ± 15.5	108.6 ± 17.2	t = 3.001	0.003
Pro–sup arc °	151.6 ± 18.9	146.4 ± 21.6	t = 1.558	0.112
Return to work/ADL by 6 months	71 (78.0)	58 (67.4)	χ² = 2.534	0.121
Patient-rated pain at 6 months (0-10)	2 (1-3)	3 (2-4)	U = 3,082	0.023

Postoperative complications were generally infrequent and comparable between groups. Rates of superficial surgical site infection (4.4% vs 8.1%, p = 0.312), deep infection (1.1% vs 2.3%, p = 0.651), iatrogenic nerve palsy (1.1% vs 4.7%, p = 0.221), heterotopic ossification (8.8% vs 15.1%, p = 0.109), fixation failure (2.2% vs 5.8%, p = 0.217), and stiffness requiring manipulation under anesthesia (3.3% vs 7.0%, p = 0.225) did not differ significantly between early and delayed groups. Similarly, reoperation rates (3.3% vs 9.3%, p = 0.089), deep vein thrombosis/pulmonary embolism (DVT/PE) events (0% vs 1.2%, p = 0.408), and 30-day readmissions (3.3% vs 7.0%, p = 0.215) showed no statistically significant differences (Table 4).

**Table 4:** Postoperative complications within six months among study participants SSI: surgical site infection; MUA: manipulation under anesthesia; DVT/PE: deep vein thrombosis/pulmonary embolism * patients may have >1 event

Complications*	Early (n = 91)	Delayed (n = 86)	Test statistic	p-value
	Frequency (%)			
Superficial SSI	4 (4.4)	7 (8.1)	χ²=1.083	0.312
Deep SSI	1 (1.1)	2 (2.3)	Fisher’s exact = 1.35	0.651
Iatrogenic nerve palsy	1 (1.1)	4 (4.7)	Fisher’s exact = 1.787	0.221
Heterotopic ossification	8 (8.8)	13 (15.1)	χ² = 1.683	0.109
Fixation failure	2 (2.2)	5 (5.8)	Fisher’s exact = 1.005	0.217
Stiffness requiring MUA	3 (3.3)	6 (7.0)	χ² = 1.321	0.225
Reoperation (any cause)	3 (3.3)	8 (9.3)	χ² = 2.890	0.089
DVT/PE	0 (0.0)	1 (1.2)	Fisher’s exact = 2.108	0.408
30-day readmission	3 (3.3)	6 (7.0)	χ² = 1.327	0.215

On multivariable logistic regression, early surgery was independently associated with higher odds of achieving MEPS ≥90 at six months compared to delayed surgery (adjusted OR: 1.90; 95% CI: 1.06-3.42; p = 0.032). Increasing age was inversely associated with this outcome (per 10-year increase: OR: 0.79; 95% CI: 0.62-0.98; p = 0.036). Gender, diabetes, open fracture, preoperative nerve deficit, and fracture type were not significant predictors in the adjusted model (Table 5).

**Table 5:** Multivariable logistic regression analysis for predictors of excellent functional outcome (MEPS ≥90) at six months MEPS: Mayo Elbow Performance Score; AO/OTA: Arbeitsgemeinschaft für Osteosynthesefragen/Orthopaedic Trauma Association; Preop: preoperative; OR: odds ratio; CI: confidence interval

Predictor	MEPS ≥90 at 6 months		Adjusted OR (95% CI)	p-value
	Yes (n = 120)	No (n = 57)		
Surgery				
Delayed (n = 86)	51 (59.3%)	35 (40.7%)	Reference	-
Early (n = 91)	69 (75.8%)	22 (24.2%)	1.901 (1.062-3.415)	0.032
Age (per 10-year increase)	-	-	0.788 (0.624-0.982)	0.036
Gender				
Female (n = 117)	78 (66.7%)	39 (33.3%)	Reference	-
Male (n = 60)	42 (70.0%)	18 (30.0%)	1.128 (0.622-2.031)	0.701
Comorbidity				
Other (n = 145)	101 (69.7%)	44 (30.3%)	Reference	-
Diabetes mellitus (n = 32)	19 (59.4%)	13 (40.6%)	0.642 (0.329-1.279)	0.291
Gustilo classification				
Closed (n = 163)	113 (69.3%)	50 (30.7%)	Reference	-
Open fracture (Gustilo I) (n = 14)	7 (50.0%)	7 (50.0%)	0.557 (0.265-1.146)	0.101
Preop nerve deficit				
No (n = 161)	111 (68.9%)	50 (31.1%)	Reference	-
Yes (n = 16)	9 (56.3%)	7 (43.7%)	0.493 (0.223-1.107)	0.088
Fracture type (AO/OTA)				
Radial head/neck (n = 71)	51 (71.8%)	20 (28.2%)	Reference	-
Distal humerus (n = 53)	33 (62.3%)	20 (37.7%)	0.621 (0.353-1.102)	0.101
Olecranon (n = 53)	36 (67.9%)	17 (32.1%)	0.882 (0.495-1.582)	0.673

To address the potential influence of age-related differences in bone healing and functional recovery, a sensitivity analysis was performed comparing adolescents (12-17 years, n = 19) and adults (≥18 years, n = 158). As shown in Table 6, the mean MEPS at six months was slightly higher among adolescents (89.3 ± 6.5) than adults (85.6 ± 8.7), though the difference approached but did not reach statistical significance (t = 1.98, df = 175, p = 0.052). Similarly, the mean flexion-extension arc was significantly greater in the adolescent subgroup (120.2° ± 14.6° vs. 111.4° ± 17.5°, t = 2.09, p = 0.038), suggesting slightly better early joint mobility. Pronation-supination arc also trended higher among adolescents (148.7° ± 12.9° vs. 139.8° ± 18.6°; p = 0.052). However, complication rates (5.3% vs. 9.5%; χ² = 0.42, p = 0.519) and proportions achieving excellent-good MEPS outcomes (94.7% vs. 83.5%; χ² = 1.53, p = 0.216) were comparable between groups.

**Table 6:** Sensitivity analysis by age category (adolescent vs adult) MEPS: Mayo Elbow Performance Score

Outcome measure	12–17 years (n = 19)	≥ 18 years (n = 158)	Test statistic	p-value
	Frequency (%)/mean ± SD			
MEPS at 6 months	89.3 ± 6.5	85.6 ± 8.7	t = 1.981	0.052
Flexion-extension arc °	120.2 ± 14.6	111.4 ± 17.5	t = 2.092	0.038
Pronation-supination arc °	148.7 ± 12.9	139.8 ± 18.6	t = 1.965	0.052
Complication rate	1 (5.3)	15 (9.5)	χ² = 0.417	0.519
Excellent-good MEPS ≥ 75	18 (94.7)	132 (83.5)	χ² = 1.533	0.216

## Discussion

In this prospective analysis of 177 patients with elbow fractures, early surgical intervention within 48 hours was associated with superior functional outcomes, reduced perioperative morbidity, and shorter hospital stays compared to delayed surgery beyond 48 hours. The two groups were well-matched in demographic and injury characteristics, minimizing potential confounding.

The most striking finding was the consistent advantage in functional recovery for the early surgery group. At six months, mean MEPS were significantly higher in early patients (91.7 ± 8.4 vs 88.6 ± 9.7, p = 0.011), and a greater proportion achieved MEPS ≥90 (75.8% vs 59.3%, p = 0.043). This advantage was already evident at six weeks and three months, indicating faster early recovery. These results mirror those of Liu et al. and Linker et al., who reported that prompt fixation of complex elbow injuries led to more rapid functional gains and higher long-term scores [14,15]. The physiological basis likely relates to early anatomical reduction before significant periarticular fibrosis and capsular contracture develop, allowing earlier initiation of mobilization protocols [16].

Range of motion findings support this mechanism: the early group achieved a greater flexion-extension arc at six months (115.8° ± 15.5 vs 108.6° ± 17.2, p = 0.003), though pronation-supination arcs were comparable (p = 0.112). Carlock et al. and Siebenbürger et al. demonstrated a similar pattern, with each day’s delay in surgery reducing the final flexion-extension arc by approximately 3°, an effect attributed to progressive soft-tissue stiffness and muscle shortening during immobilization [17,18]. Our finding that pronation-supination was unaffected may reflect the relative preservation of forearm rotation even with periarticular scarring, compared to the greater capsular involvement in flexion-extension [19].

Pain scores at six months were also lower in the early group (median 2 vs 3, p = 0.023), consistent with reports by Dickens et al., that early fixation reduces chronic discomfort, likely through minimizing prolonged inflammation and abnormal joint loading [20]. While return-to-work or ADL rates did not differ significantly (p = 0.121), earlier pain reduction and functional recovery may still translate into better quality-of-life outcomes over a longer horizon [21].

From a perioperative standpoint, early surgery offered measurable efficiency benefits: shorter operative times (94.5 ± 28.9 vs 102.7 ± 32.9 min, p = 0.004), reduced intraoperative blood loss (163.9 ± 82.6 vs 199.5 ± 91.6 mL, p = 0.042), and less fluoroscopy time (78.7 ± 25.4 vs 86.7 ± 28.3 s, p = 0.046). Ma et al. and Cañada-Oya et al. similarly found that operating within 48 hours reduced technical complexity, attributing this to less soft-tissue swelling, better visualization of fracture planes, and easier fragment manipulation [22,23]. Shorter immobilization (median 2 vs 3 days, p = 0.011) and earlier physiotherapy initiation (93.4% vs 79.1%, p = 0.007) in the early group likely compounded these advantages, facilitating a virtuous cycle of recovery [24].

Hospital stay was shorter for early patients (median 4 vs 5 days, p = 0.014; mean 4.8 ± 1.2 vs 6.1 ± 1.4 days, p < 0.001), in line with meta-analyses by Zhao et al. and more recent Indian cohort studies, which consistently report 1-2 days’ reduction in acute care needs when surgery is not delayed [25]. Shorter stays not only reduce healthcare costs but also limit nosocomial exposure and allow earlier community reintegration [26].

Overall complication rates were lower in the early group (12.1% vs 25.6%, p = 0.021), particularly for infection and stiffness. Although individual complication subtypes did not reach statistical significance, trends favored early surgery for most outcomes. This supports the hypothesis that timely intervention minimizes the combined risks of wound breakdown from tense, swollen tissues and postoperative arthrofibrosis from prolonged immobilization [26]. Interestingly, rates of reoperation, heterotopic ossification, and iatrogenic nerve palsy were not significantly different, suggesting that these may be more influenced by fracture severity and surgical technique than timing alone [27,28].

Multivariable logistic regression confirmed early surgery as an independent predictor of excellent/good MEPS outcomes at six months (adjusted OR: 1.90; 95% CI: 1.06-3.42; p = 0.032), even after adjusting for age, comorbidities, fracture type, and injury severity. Increasing age was a negative predictor (OR: 0.79 per decade; p = 0.036), echoing prior findings that diminished bone quality, slower healing, and less aggressive rehabilitation in older patients can blunt surgical gains regardless of timing [29,30].

Clinical implications

In the context of Indian tertiary care hospitals, the findings underscore the importance of adopting standardized protocols that prioritize early fixation, preferably within 48 hours of injury, for improved functional recovery and reduced stiffness in elbow fractures. The demonstrated benefits in range of motion and early return to activities suggest that timely surgery could become a key quality indicator in orthopedic trauma care. Achieving this would require the integration of streamlined emergency trauma pathways, strategic allocation of operating room slots, and administrative support to facilitate earlier surgical scheduling, even during peak caseloads. However, it is equally important to recognize the influence of case complexity and patient-specific factors. Institutional constraints, concurrent life-threatening injuries in polytrauma patients, or soft-tissue conditions such as significant swelling or compromised skin integrity may necessitate individualized surgical timing. Hence, while early fixation is beneficial in most scenarios, clinical judgment should remain central to decision-making.

Limitations and future directions

This single-center study may limit generalizability due to variations in institutional protocols and patient demographics. The moderate sample size constrained subgroup analyses, particularly for age and fracture type. The relatively short follow-up duration of six months might not adequately capture late complications such as posttraumatic arthritis or hardware failure. Additionally, radiographic parameters were not correlated with functional outcomes, and validated patient-reported outcome measures, such as the disabilities of the arm, shoulder, and hand (DASH) or the patient-rated wrist evaluation (PRWE), were not incorporated. Variations in surgical timing could partly reflect logistical or clinical prioritization factors that might have influenced outcomes.

Furthermore, the absence of randomization introduces the possibility of residual confounding despite multivariable regression adjustment. Future studies should adopt multicenter randomized controlled designs with larger, demographically diverse cohorts and extended follow-up durations. Incorporating standardized radiological and patient-reported metrics would enable a more comprehensive assessment of recovery. Evaluating cost-effectiveness and workflow optimization of early fixation protocols could guide policy and implementation in resource-limited trauma systems.

## Conclusions

Early surgical intervention for elbow fractures, particularly within 24-48 hours of injury, was associated with significantly better functional outcomes, reduced postoperative stiffness, and shorter rehabilitation time compared to delayed surgery. While overall complication rates were similar, early surgery demonstrated fewer infections and transient neuropraxia, reinforcing the benefits of timely fixation. These findings support prioritizing early operative management in suitable candidates to enhance functional recovery and patient satisfaction. Future integration of structured early fixation pathways within trauma care systems may further improve patient throughput, reduce hospital stay, and enhance cost-efficiency across tertiary orthopedic centers.
